# Restoration Method of a Blurred Star Image for a Star Sensor Under Dynamic Conditions

**DOI:** 10.3390/s19194127

**Published:** 2019-09-24

**Authors:** Zhiya Mu, Jun Wang, Xin He, Zhonghui Wei, Jiawei He, Lei Zhang, You Lv, Dinglong He

**Affiliations:** Changchun Institute of Optics, Fine Mechanics and Physics, Chinese Academy of Sciences, Changchun 130033, China; muziya9@163.com (Z.M.); hexin6627@sohu.com (X.H.); wzhlvp@sohu.com (Z.W.); hejiawei3650@163.com (J.H.); leo_zh.l@ailiyun.com (L.Z.); lvyou8863@163.com (Y.L.); 18843096680@163.com (D.H.)

**Keywords:** star sensor, motion blur, curvature filter, image restoration

## Abstract

Under the dynamic working conditions of a star sensor, motion blur of the star will appear due to its energy dispersion during imaging, leading to the degradation of the star centroid accuracy and attitude accuracy of the star sensor. To address this, a restoration method of a blurred star image for a star sensor under dynamic conditions is presented in this paper. First, a kinematic model of the star centroid and the degradation function of blurred star image under different conditions are analyzed. Then, an improved curvature filtering method based on energy function is proposed to remove the noise and improve the signal-to-noise ratio of the star image. Finally, the Richardson Lucy algorithm is used and the termination condition of the iterative equation is established by using the star centroid coordinates in three consecutive frames of restored images to ensure the restoration effect of the blurred star image and the accuracy of the star centroid coordinates. Under the dynamic condition of 0~4°/s, the proposed algorithm can effectively improve the signal-to-noise ratio of a blurred star image and maintain an error of the star centroid coordinates that is less than 0.1 pixels, which meets the requirement for high centroid accuracy.

## 1. Introduction

A star sensor is an optical device that measures the positions of stars to determine attitude or orientation. This kind of instrument has been widely used in space missions and deep space exploration, allowing high precision astronomical navigation. A star sensor works by first imaging the starry sky, and then determining the star centroid coordinate information of stars using star extraction and location methods. Once this information has been obtained, the star positions can be compared with the known absolute positions from a star catalog. Finally, based on this comparison, the three-axis attitude of the star sensor relative to the inertial coordinate system can be calculated. Generally, the star sensor works under static condition and it is typically used during the steady flight state of the carrier. It is assumed that the navigation star and the star sensor are relatively stationary during the exposure time, and the star point is imaged at a fixed position on the image plane. When the star sensor is implemented under dynamic conditions, during exposure time, the star forms a trajectory image on the image plane, leading to the degradation of the signal-to-noise ratio (SNR) and decreased accuracy of the star centroid, which may even prevent extraction of the star position information, thus affecting the overall attitude accuracy of the star sensor.

Therefore, improving dynamic performance is needed to improve the performance of the star sensor. The key focus of efforts to improve the dynamic performance of the star sensor is to optimize the denoising and restoration algorithms for blurred star images. 

Many scholars have proposed denoising and restoration algorithms of blurred star images. Bezooijen et al. [1] proposed a time-delayed integration (TDI) method. In this method, the motion blur of the star was reduced using a special hardware sequential circuit, which improved the SNR of image. However, this method only eliminated motion blur in the y-direction, so an image-processing algorithm is required to further improve the SNR. Based on the TDI method, Pasetti et al. [2] compensated for the effect of motion by oversampling and binning. Sun et al. [3] established a motion model of the star centroid by using the angular velocity provided by a gyro, then the degradation function of the blurred star image was obtained and the Richardson Lucy (RL) algorithm [4,5] was used to restore the blurred star image. However, the angular velocity provided by the gyro drifts with time, which can also seriously affect the accuracy of the determination of the star centroid. To address this, Sun et al. [6] revised the gyro data by applying an Extended Kalman filter to improve the accuracy of the motion model. Ma et al. [7] proposed a multi-seed-region growing technique to preprocess the star image before restoration, but the size of the filtering template was limited, which caused a loss of star energy. Zhang et al. [8] denoised the blurred star image by using adaptive wavelet thresholding, and then restored the star image with an improved Wiener filter. Sun et al. [9] removed background noise by correlation filtering and morphological filtering, then determined the angle and length of the blurred star by using an image differential method and the star centroid was calculated. However, these methods obtained the star centroid from a blurred star image, which limited the accuracy.

Given the shortcomings of present methods for the denoising and restoration of a blurred star image, we propose a restoration method for a blurred star image under dynamic conditions. In this approach, the kinematic model of the star centroid and the degradation function of a blurred star image under different conditions are first analyzed. Then, an improved curvature filtering method based on energy function is utilized to remove the noise and improve the SNR of the star image. Finally, the termination condition of the iterative equation in the RL algorithm is established by using the star centroid coordinates obtained from three consecutive frames of restored images, allowing good restoration effect of the blurred star image and high accuracy of the star centroid determination.

## 2. Parameter Estimation of Blurred Star Image

### 2.1. Motion Model of Star Centroid

**Figure 1:**
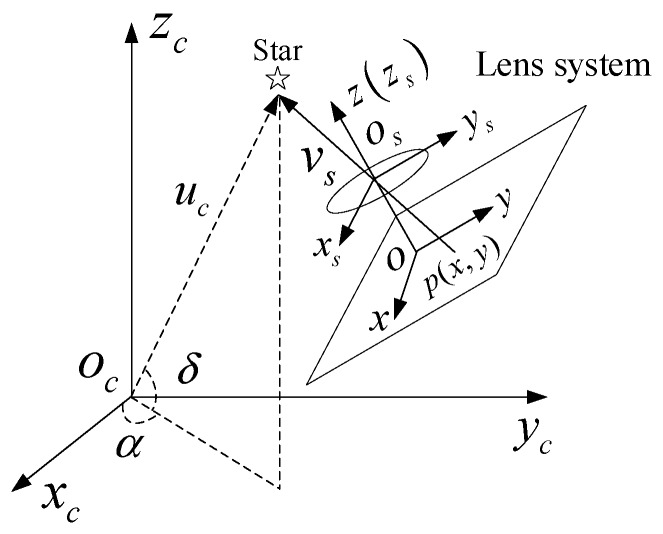
The attitude measurement model of a star sensor.

A star sensor is an attitude-measuring instrument that offers measurement accuracy of angular second [10]. The attitude measurement model is shown in Figure 1.

In the inertial coordinate system $o_{c}-x_{c}y_{c}z_{c}$, the reference unit vector $u_{c}$ of the star point can be expressed using the right ascension α and declination δ as Equation (1).

(1) $$u_{c}=\left[\begin{array}{c} \cos\alpha \cos\delta \\ \sin\alpha \cos\delta \\ \sin\delta \end{array}\right],$$

In the image space coordinate system o−xyz, the observation unit vector $v_{s}$ of the star point can be expressed from the coordinate p(x,y) of the star point on the image plane and the focal length f of the optical system, and Equation (2) can be obtained.

(2) $$v_{s}=\frac{1}{\sqrt{x^{2}+y^{2}+f^{2}}}\left[\begin{array}{c} -x\\ -y\\ f \end{array}\right],$$

Theoretically, the reference unit vector $u_{c}$ and the observation unit vector $v_{s}$ satisfy the following equality relations:

(3) $$v_{s}=Au_{c},$$

In Equation (3), A is the rotation matrix between the image space coordinate system and the inertial coordinate system, which is also called the attitude matrix of the star sensor. The attitude matrix can be used to calculate the attitude of the star sensor with respect to the three-axis attitude of the inertial coordinate system.

It is assumed that at $t_{0}$ time, the centroid coordinates of the stars on the image plane are $\left(x(t_{0}),y(t_{0})\right)$, and the corresponding observation unit vector is $v_{s}(t_{0})$. At $t_{0}+\Delta t$ time (Δt≪T, where T is the star sensor exposure time, usually in milliseconds), the centroid coordinates of the star point are $\left(x(t_{0}+\Delta t),y(t_{0}+\Delta t)\right)$, and the corresponding observation unit vector is $v_{s}(t_{0}+\Delta t)$. Then, Equation (4) can be obtained from Equation (3).

(4) $$\{\begin{array}{l} v_{s}(t_{0})=A(t_{0})u_{c}\\ v_{s}(t_{0}+\Delta t)=A(t_{0}+\Delta t)u_{c} \end{array},$$

At the time of $t_{0}$ and $t_{0}+\Delta t$, the star points have the same reference unit vector $u_{c}$ in the inertial coordinate system, so:

(5) $$v_{s}(t_{0}+\Delta t)=A_{t_{0}}^{t_{0}+\Delta t}v_{s}(t_{0}),$$

In Equation (5), $A_{t_{0}}^{t_{0}+\Delta t}=A(t_{0}+\Delta t)A^{-1}(t_{0})$ is the attitude transfer matrix from $t_{0}$ to $t_{0}+\Delta t$ of the star sensor, and the Taylor expansion of the attitude transfer matrix is

(6) $$A_{t_{0}}^{t_{0}+\Delta t}=I_{3\times 3}-(w\times )\cdot \Delta t+\frac{1}{2}[ww^{T}-w^{T}w\cdot I_{3\times 3}-(w\times )]\cdot {\left(\Delta t\right)}^{2}+\varepsilon ({\left(\Delta t\right)}^{2}),$$

Since the time interval Δt is short, the term multiplied by ${\left(\Delta t\right)}^{2}$ and its higher order terms can be neglected, so the attitude transfer matrix can be written as Equation (7).

(7) $$A_{t_{0}}^{t_{0}+\Delta t}\approx I_{3\times 3}-(w\times )\cdot \Delta t=\left[\begin{array}{ccc} 1 & w^{z}\Delta t & -w^{y}\Delta t\\ -w^{z}\Delta t & 1 & w^{x}\Delta t\\ w^{y}\Delta t & -w^{x}\Delta t & 1 \end{array}\right],$$

In Equation (7), $w={\left[w_{x}\;w_{y}\;w_{z}\right]}^{\;T}$ is the angular velocity vector of the star sensor, which is usually supplied by the inertial navigation system, and w× is the three-order square matrix that is orthogonal to w.

It is assumed that the angular velocity of the star sensor remains almost unchanged within a very short time interval of Δt, so the motion model (Equation (8)) of the centroid of the star point from $t_{0}$ to $t_{0}+\Delta t$ can be obtained by Equation (5).

(8) $$\{\begin{array}{l} x(t_{0}+\Delta t)=\frac{x(t_{0})+y(t_{0})w^{z}\Delta t+fw^{y}\Delta t}{1-\left[x(t_{0})w^{y}\Delta t-y(t_{0})w^{x}\Delta t\right]/f}\\ y(t_{0}+\Delta t)=\frac{y(t_{0})-x(t_{0})w^{z}\Delta t-fw^{x}\Delta t}{1-\left[x(t_{0})w^{y}\Delta t-y(t_{0})w^{x}\Delta t\right]/f} \end{array},$$

According to the parameters of the star sensor in this paper, the pixel size is in micron level, the focal length is in millimeter level, and the exposure time is in millisecond level, hence $\left[x(t_{0})w^{y}\Delta t-y(t_{0})w^{x}\Delta t\right]/f\ll 1$ is established, so the motion model of star centroid can be approximately expressed as Equation (9).

(9) $$\{\begin{array}{l} x(t_{0}+\Delta t)=x(t_{0})+y(t_{0})w^{z}\Delta t+fw^{y}\Delta t\\ y(t_{0}+\Delta t)=y(t_{0})-x(t_{0})w^{z}\Delta t-fw^{x}\Delta t \end{array},$$

### 2.2. The Degenerate Function of a Motion Blurred Image

Using Equation (9), we can analyze the motion model of the star centroid and the degeneration function of the blurred star image under two different working conditions—one in which the star sensor rotates only around the Z axis and one in which the star sensor rotates around the X axis and the Y axis at the same time.

(1) Star sensor rotates only around the Z axis

When the star sensor only rotates around the Z axis, the angular velocity of the X axis and the Y axis satisfies $w_{x}=w_{y}=0$, and is substituted in Equation (9).

(10) $$\{\begin{array}{l} x(t_{0}+\Delta t)=x(t_{0})+y(t_{0})w^{z}\Delta t\\ y(t_{0}+\Delta t)=y(t_{0})-x(t_{0})w^{z}\Delta t \end{array},$$

From Equations (10) and (11) can be obtained:

(11) $$x^{2}(t)+y^{2}(t)=c^{2},$$

In Equation (11), $c=\sqrt{x^{2}(t_{0})+y^{2}(t_{0})}$. Therefore, when the star sensor rotates only around the Z axis, in a very short time interval of Δt, the motion model of the centroid of the star point is a segment of arc $\overset{⌢}{l_{z}}$ in a circle with a radius of c. The degenerate function of the motion blurred star image is as Equation (12).

(12) $$h_{z}(x,y)=\{\begin{array}{cc} 1/cw^{z}\Delta t & p(x,y)\in \overset{⌢}{l_{z}}\\ 0 & otherwise \end{array},$$

**Figure 2:**
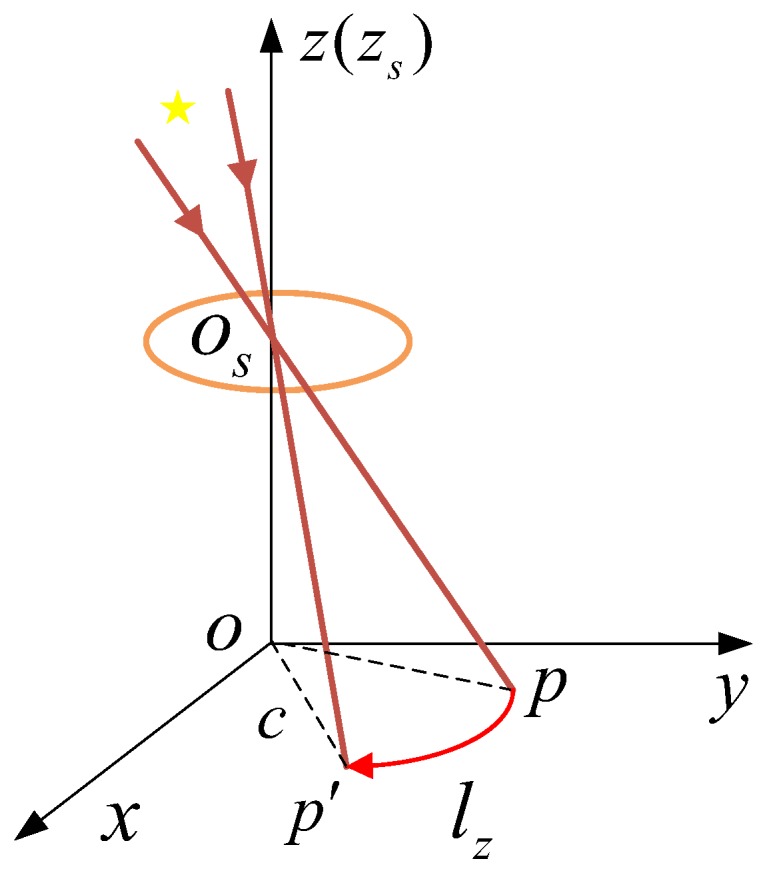
Model of star centroid when rotating around Z axis.

According to Equation (11), the model of the star centroid is shown in Figure 2.

(2) The star sensor rotates around the X axis and the Y axis simultaneously

When the star sensor rotates around the X axis and the Y axis simultaneously, the angular velocity of the Z axis satisfies $w_{z}=0$, and can be substituted in Equation (9).

(13) $$\{\begin{array}{l} x(t_{0}+\Delta t)=x(t_{0})+fw^{y}\Delta t\\ y(t_{0}+\Delta t)=y(t_{0})-fw^{x}\Delta t \end{array},$$

From Equations (13) and (14) can be obtained:

(14) $$\frac{y(t_{0}+\Delta t)-y(t_{0})}{x(t_{0}+\Delta t)-x(t_{0})}=-\frac{w^{x}}{w^{y}},$$

Therefore, when the star sensor rotates around the X axis and the Y axis at the same time, the motion model of the centroid of the star point is a line segment $l_{xy}$ with a length of $L_{xy}=f\Delta t\sqrt{{\left(w^{y}\right)}^{2}+{\left(w^{x}\right)}^{2}}$ and the slope of $k=-w^{x}/w^{y}$. Thus, the degeneration function of the motion blurred star image can be expressed as Equation (15).

(15) $$h_{xy}(x,y)=\{\begin{array}{ll} 1/L_{xy} & p(x,y)\in l_{xy}\\ 0 & otherwise \end{array},$$

**Figure 3:**
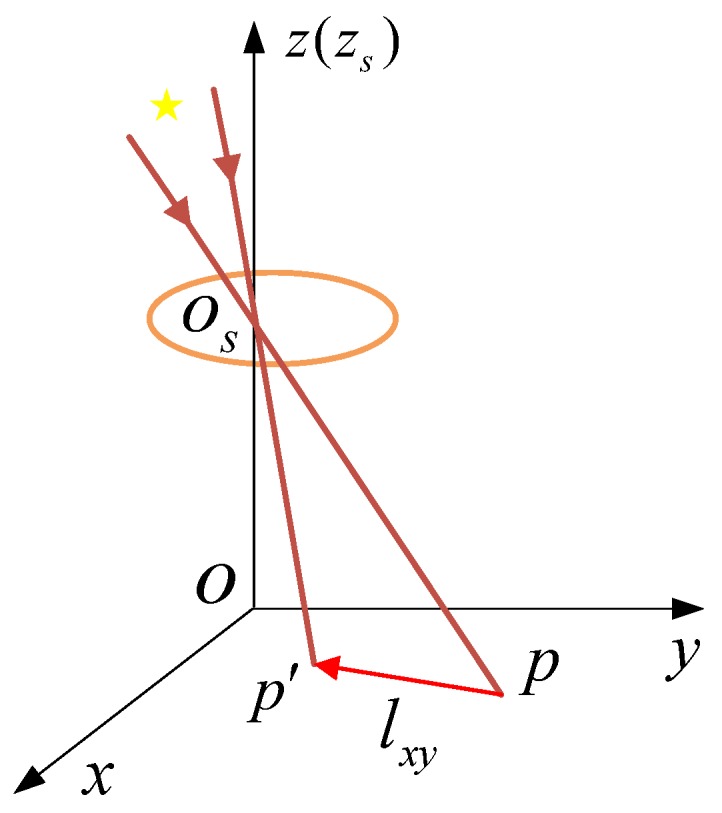
Model of star centroid when rotating around the X and Y axes.

According to Equation (14), the model of star centroid is shown in Figure 3.

## 3. Blurred Star Image Denoising

**Figure 4:**
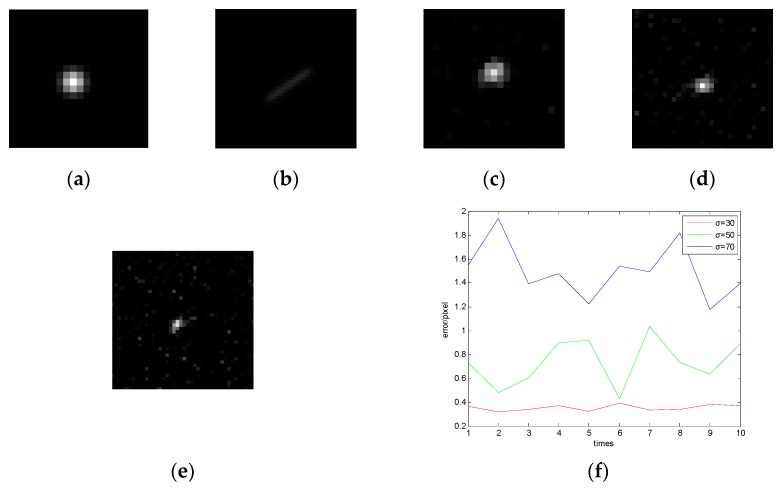
Effect of noise on centroid extraction after restoration. (**a**) Original image; (**b**) star image under dynamic conditions; (**c**) result of Richardson Lucy (RL) restoration with variance of 30; (**d**) result of RL restoration with variance of 50; (**e**) result of RL restoration with variance of 70; (**f**) error curve of the X axis.

The original star image contains a significant amount of additive noise, which includes salt and pepper noise, Gauss white noise, and Poisson noise [11]. Additive noise is unrelated to the original information of the image, but destroys the image signal by superposition. When a star sensor works for a long period of time, there will be electronic thermal noise and high frequationuency electromagnetic interference noise in the image. These noises have the characteristics of high energy and random distribution, and are difficult to estimate. In this study, the Gauss model is used to construct an original star image, based on which a blurred star image is obtained under the dynamic condition of $w_{x}=5^{\circ }/s,w_{y}=3.5^{\circ }/s$ according to the parameters of Table 1, and Gauss noise with mean value of 0 and variance of 30, 50, and 70 are added to it, respectively. Then the traditional RL algorithm is used to restore the blurred star image with different noise for 100 times, and then the star centroid coordinates in the star images can be calculated. Finally, the average value of every 10 star centroid coordinates is obtained, and the star centroid error can be obtained through the comparison with the true star centroid coordinate. The error curves (considering the X axis as an example) are shown in Figure 4.

Figure 4c–e shows that the larger the noise is, the worse the restoration effect is, because when noise exists in the star image, the RL algorithm will amplify the noise. Besides, once the noise is too large, the star will be submerged, resulting in a significant decrease in the accuracy of the star centroid. Therefore, denoising of the blurred star image is requationuired to achieve a highly accurate star centroid location.

λ=(i,j)∈Ω represents the spatial coordinate of the image. We use U(λ) to represent the current reconstructed image, and $U_{xx}$, $U_{yy}$, $U_{x}$, $U_{y}$ are the partial derivatives, so the image curvature can be described as:

(16) $$K(U(\lambda ))=\frac{U_{xx}U_{yy}-U_{xy}^{2}}{{\left(1+U_{x}^{2}+U_{y}^{2}\right)}^{2}},$$

To avoid the complexity of the explicit calculation of Gauss curvature, Gong [12] assumed that the surface of the original image was piecewise developable, which meant the Gauss curvature was zero everywhere, allowing a good image smoothing effect with edge protection. To meet the assumption that the image is piecewise developable, the gray value of each pixel is directly adjusted to the tangent plane of the neighboring pixels, and then the curvature information of the image is implicitly used to protect the details of the image. The method uses the principle of minimum distance adjustment, which corrects the gray value using the surface closest to the current pixel among all the tangent planes composed of neighboring pixels.

**Figure 5:**
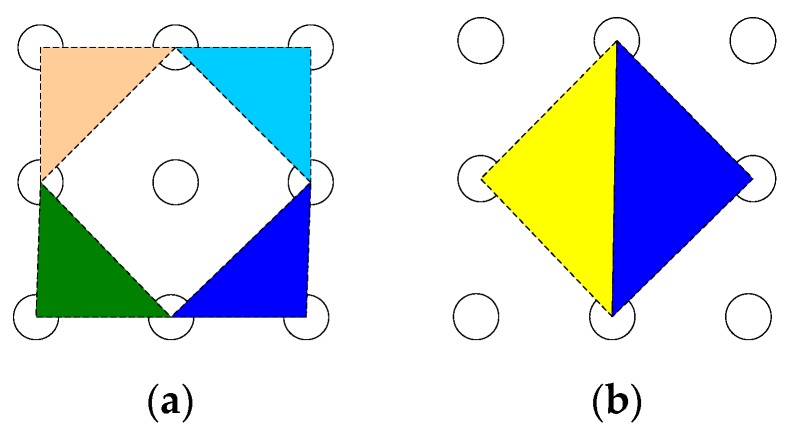
Basic projection operator diagram. (**a**) Diagonal tangent planes; (**b**) minimum triangular tangent planes.

The Gauss curvature filter has a good effect in dealing with Gauss, salt and pepper noise. The Gauss curvature filter requationuires several iterations to eliminate the noise and single iteration is generally insufficient. However, multiple iterations may smooth the star excessively, which will affect the energy distribution of the star and have a great impact on the image restoration. Therefore, an improved Gauss curvature filtering method based on energy function is proposed in this paper to denoise the image. In the algorithm, the distance between the pixel (x,y) and the tangent plane formed by the neighboring pixels in the 3 × 3 window should be calculated. As shown in Figure 5, the neighborhoods are divided into four kinds of diagonal tangent planes and four kinds of minimum triangular tangent planes, and the combination of diagonal tangent planes and minimum triangular tangent planes are used as basic projection operators, the advantages of which are as follows. First, the diagonal tangent plane enhances the connection of neighboring pixels and can effectively suppress the Gauss speckle noise in star images. Second, the minimum triangular tangent plane has a better effect on the salt and pepper noise.

(17) $$\begin{array}{l} d_{1}=(f(x-1,y)+f(x+1,y)+f(x,y+1))/3-f(x,y)\\ d_{2}=(f(x,y-1)+f(x+1,y-1)+f(x+1,y))/3-f(x,y)\\ d_{3}=(f(x-1,y-1)+f(x-1,y)+f(x,y-1))/3-f(x,y)\\ d_{4}=(f(x,y+1)+f(x+1,y)+f(x+1,y+1))/3-f(x,y)\\ d_{5}=(f(x,y-1)+f(x,y+1)+f(x+1,y))/3-f(x,y)\\ d_{6}=(f(x-1,y)+f(x,y+1)+f(x-1,y+1))/3-f(x,y)\\ d_{7}=(f(x,y-1)+f(x,y+1)+f(x-1,y))/3-f(x,y)\\ d_{8}=(f(x-1,y)+f(x,y-1)+f(x+1,y))/3-f(x,y) \end{array}$$

In Equation (17), f(x,y) denotes the gray value at (x,y), $d_{n}$ represents the distance between (x,y) and the tangent plane of its neighboring point, then the gray value of the pixel is corrected using the minimum distance adjustment method.

(18) $$\hat{f}(x,y)=f(x,y)+d_{m}\;\left|d_{m}\right|=\min\{\left|d_{n}\right|,n=1\cdots 8\},$$

In Equation (18), $\hat{f}(x,y)$ represents the corrected gray value at (x,y). By using a sliding window to filter each pixel of the image, the Gauss curvature filter of the image can be completed.

To solve the uneven energy distribution of the star caused by the multiple iterations of the curvature filter, a new energy model is established based on the energy minimization theory to distinguish the star and noise. Then, the improved curvature filter is used to obtain the final estimated gray value of the star and the energy of the star contaminated by the noise can be estimated and restored.

In this paper, the energy function of the pixel is defined as E(x,y), which can represent the difference between the central pixel and the surrounding pixels in the neighborhood of the image. Besides, $E_{s}(x,y)$ is the potential energy of the pixel (x,y), $E_{d}(x,y)$ is the energy value between the central pixel and other pixels in the neighborhood, and v(x,y) are the eight neighborhoods of the pixel (x,y). The energy function model is established as follows:

(19) $$E(x,y)=E_{s}(x,y)+E_{d}(x,y),$$

Among:

(20) $$E_{s}(x,y)=\left|f(x,y)-\hat{f}(x,y)\right|,$$

(21) $$E_{d}(x,y)=\sum_{k=1}^{4}r_{k},$$

(22) A(x,y)=φ(f(x,y)−f(k,l))k,l∈v(x,y),

(23) $$\varphi (t)={\left|t\right|}^{\alpha }\;1\leq \alpha \leq 2,$$

**Figure 6:**
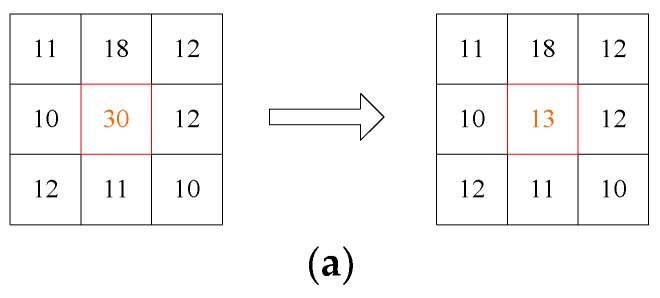

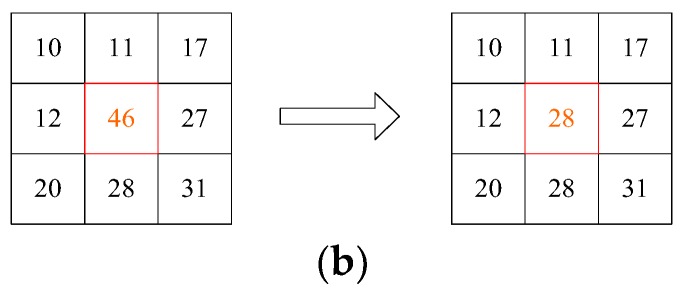
Denoising algorithm in this paper. (**a**) Diagram of noise removal; (**b**)diagram of restoring the energy of the star.

In Equation (20), $\hat{f}(x,y)$ is the estimated value by the Gaussian curvature filter. In Equations (21)–(23), φ(t) is the edge-preserving potential function, A(x,y) is the potential function between the central pixel and surrounding pixels, $r_{k}$ is the kth smallest A(x,y) in the neighborhood, and $E_{d}(x,y)$ is the sum of four smallest $r_{k}$ values. Generally, the gray values of the noise-contaminated pixels are not continuous with that of the neighboring pixels, hence there will be a large variation in the gray values. In this paper, the specific assessment of the noise is performed using a 3 × 3 filter template to traverse the image, and then the energy value E(x,y) of each pixel is calculated. When E(x,y) is less than the threshold T, the pixel is considered as an uncontaminated signal point, otherwise the pixel is considered as a point contaminated by noise, and then the noise should be filtered. Therefore, the proposed energy function is used to distinguish the noise from the effective signal point of the star, and the superimposed noise both in the star point and the sky background are processed by the curvature filter, which can remove the noise without destroying the effective signal. As shown in Figure 6a, the central pixel is a speckle noise, and the gray value changes from 30 to 13 after being processed, which indicates that the noise is denoised successfully. In addition, the central pixel in Figure 6b is the contaminated star point, and the gray value changes from 46 to 28 after being processed, which achieves the goal of restoring the energy of the star.

## 4. Restoration of Motion Blurred Star Image

**Figure 7:**
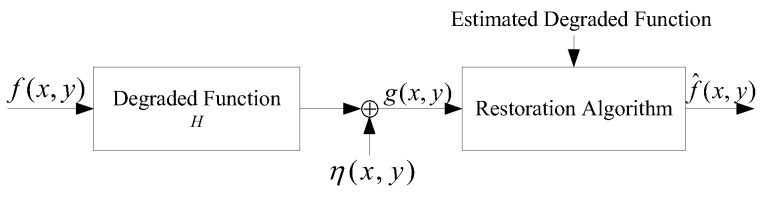
Degradation and restoration model of image.

The restoration of a blurred star image requires the establishment of a degenerate/restoration model of the image. Gonzalez [13] proposed that the degradation process of an image could be modeled using a degradation function H and an additive noise term η(x,y). An input image f(x,y) is processed to produce a degraded image g(x,y). If g(x,y), the degradation function H and the additive noise term η(x,y) are known, then an estimated $\hat{f}(x,y)$ of the original image can be obtained. The degradation/restoration model of the image is shown in Figure 7.

In this paper, RL algorithm is used to restore the blurred star image. The RL algorithm is a classical algorithm for image restoration, which assumes that the image obeys Poisson distribution and can be estimated by the maximum likelihood method. It is an iterative algorithm based on Bayesian analysis [14], which requires little prior knowledge and provides good restoration effect. Its iterative equation is presented in Equation (24).

(24) $$f^{\left(k+1\right)}=f^{\left(k\right)}\left[\left(\frac{g}{f^{\left(k\right)}*h}\right)⊕h\right],$$

In Equation (24), ∗ represents the convolution operation, ⊕ represents the correlation operation, and g is a blurred star image. h is the convolution operation result of Equations (12) and (15), where $h(x,y)=h_{z}(x,y)*h_{xy}(x,y)$. $f^{\left(k\right)}$ and $f^{\left(k+1\right)}$ are the reconstructed images after *k* and *k* + 1 iterations, respectively.

It can be seen from Equation (24) that there is no termination condition in the iterative process of the RL algorithm, so the iteration number k must be selected based on experience. If k is too small, the blurred star image will not be fully restored and the result of the star location cannot meet the requirement of high accuracy. In contrast, if k is too large, the processing time of the blurred star image will increase, and it not only reduces the update rate of the star sensor, but also causes the amplification of noise in the iteration process, which seriously affects the accuracy of the star centroid [15]. To solve the problem, we establish the termination condition of the iterative equation by using the star centroid coordinates in three consecutive frames of restored images and the parameter of the blurred star image.

(25) $$\{\begin{array}{c} \left|\left|x_{k+1}-x_{k}\right|-\left|x_{k}-x_{k-1}\right|\right|\leq \varepsilon (\Delta x)\\ \left|\left|y_{k+1}-y_{k}\right|-\left|y_{k}-y_{k-1}\right|\right|\leq \varepsilon (\Delta y) \end{array},$$

In Equation (25), $\left(x_{k-1},y_{k-1}\right)$, $\left(x_{k},y_{k}\right)$, and $\left(x_{k+1},y_{k+1}\right)$ are the star centroid coordinates in the restored images after k−1, k and k+1 iterations. Δx and Δy are the blurred length in the x and y directions, respectively. ε(Δx) and ε(Δy) are the functions about Δx and Δy, which should be selected appropriately after weighing the efficiency and effect of restoration. By using the proposed algorithm, the iterative process can be stopped once the restored star image satisfies the termination condition.

## 5. Results and Analysis

**Figure 8:**
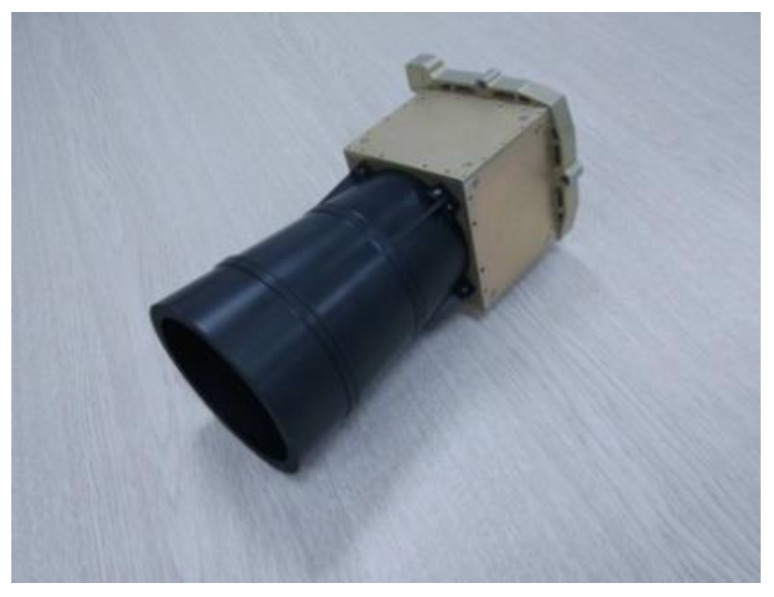
Star sensor in simulation.

This section is divided by subheadings. It should provide a concise and precise description of the experimental results, their interpretation, as well as the experimental conclusions that can be drawn. The simulations are implemented with MATLAB in Windows operating system on a Core VIII computer with 3.2 GHz frequency. The star sensor used in the simulations is shown in Figure 8, and its detailed parameters are listed in Table 1. The angular velocity of the star sensor is 0~4°/s.

### 5.1. Denoising of the Blurred Star Image

**Figure 9:**
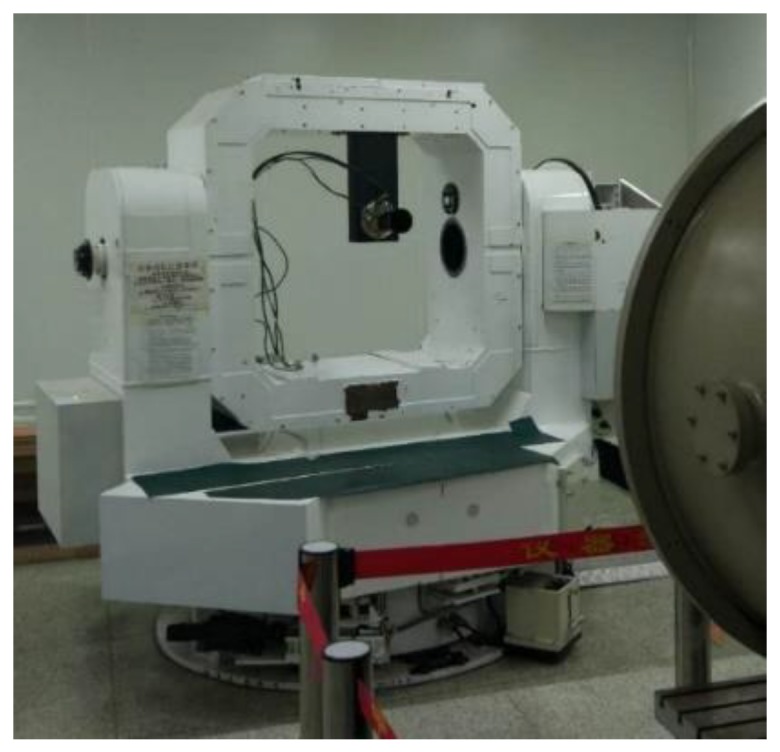
Experimental platform of star sensor.

**Figure 10:**
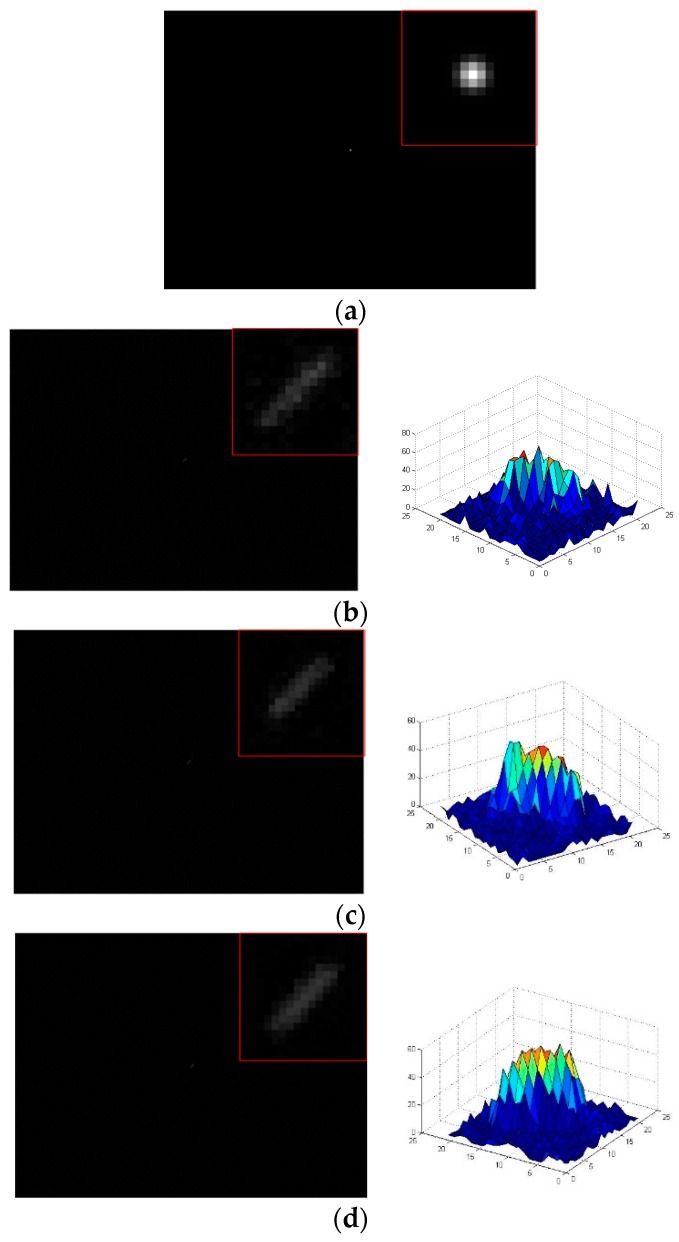

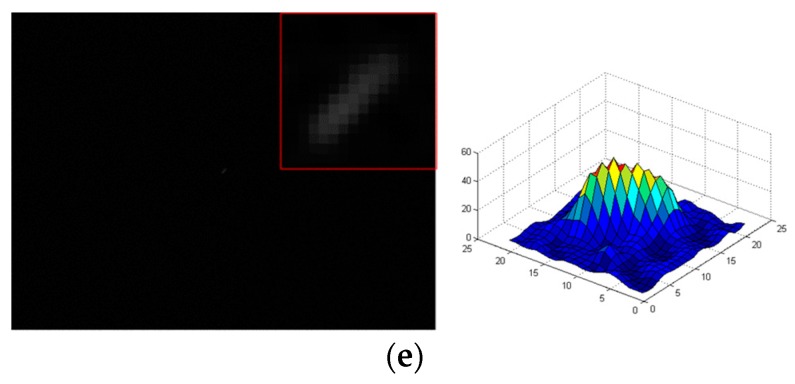
Denoised star images with different methods. (**a**) Star image under static condition; (**b**) blurred star image with a noise covariance of 50 and its three-dimensional distribution; (**c**) denoised star image with Bayes Shrink and its three-dimensional distribution; (**d**) denoised star image with open operation and its three-dimensional distribution; (**e**) denoised star image with the proposed algorithm and its three-dimensional distribution.

As shown in Figure 9, the experimental platform is comprised of a two-dimensional turntable of high precision and a parallel light pipe, and the actual star image obtained by the star sensor is shown in Figure 10a. Under the dynamic condition of $w_{x}=w_{y}=4^{\circ }/s$, the star image in Figure 10a is blurred according to the parameters of star sensor, and Gaussian noise with variances of 30, 50, and 70 are added into the star image, respectively. Then, the Bayes Shrink method [16], open operation [17], and the proposed algorithm are applied to denoise the star image. The blurred star image with a noise variance of 50 is shown in Figure 10b. By comparison with Figure 10a, it is obvious that under dynamic condition, the energy of the star is dispersed and contaminated by noise, which results in a reduced gray level of the star. As shown in Figure 10a–e, by using the proposed algorithm, the energy of the star in the denoised star image is more uniform and closer to the Gaussian distribution; however, the denoising effect of other methods is not obvious and the energy of the star is not uniform.

To further verify the denoising performance of the proposed algorithm, Figure 10a is blurred under the dynamic condition of $w_{x}=w_{y}=1^{\circ }/s$, $w_{x}=w_{y}=2^{\circ }/s$, and $w_{x}=w_{y}=4^{\circ }/s$, respectively. Then, the Gaussian noise with variances of 30, 50, and 70 are independently added into the blurred star images, and the Bayes Shrink method, open operation, and the proposed algorithm are used to denoise the star images. The signal-to-noise ratios (PSNRs) of the denoised star images are obtained and shown in Table 2. The experimental results indicate that with increased angular velocity, the denoising effect and the improvement of PSNR of the proposed algorithm are better than that of the other two methods. Under the same angular velocity, when the variance of noise grows, the proposed algorithm can maintain better denoising effect.

**Figure 11:**
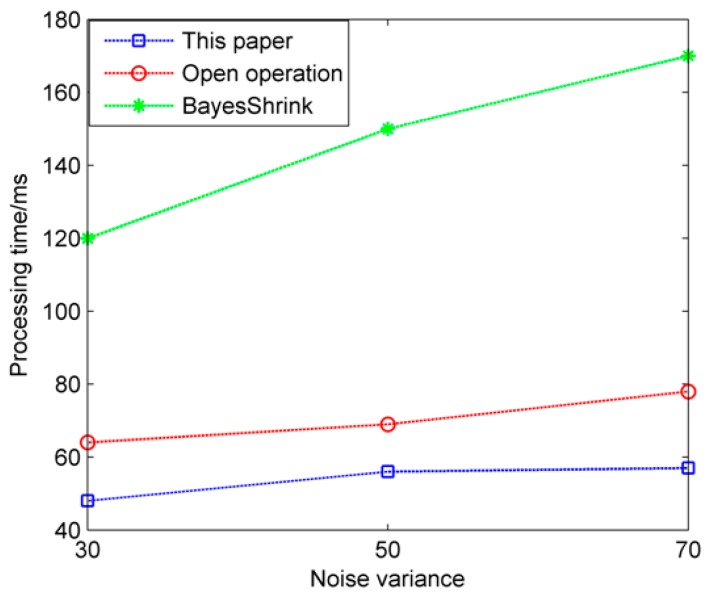
Processing time of denoising algorithms.

In order to verify the processing speed of the denoising algorithm in this paper, we record the processing time of the three algorithms under different noise variances in the process of denoising experiments, and the results are shown in Figure 11. As shown in Figure 11, the processing speed of the proposed algorithm is significantly faster than that of Bayes Shrink and open operation under different noise variances.

**Figure 12:**
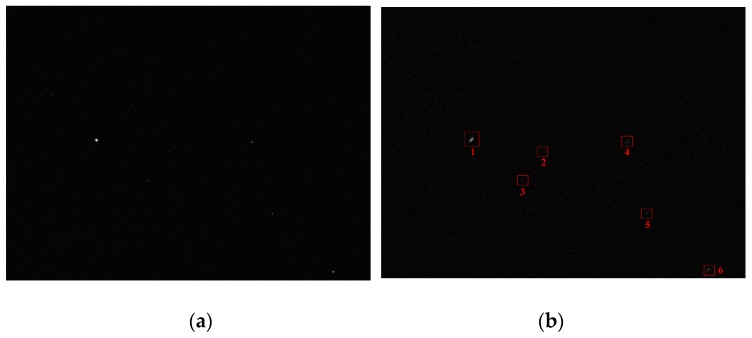

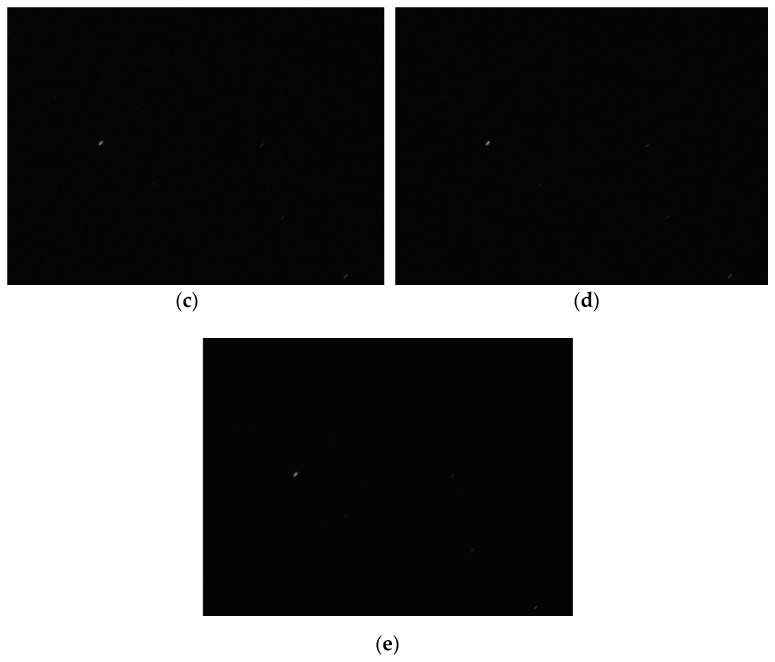
Denoised star images with different methods. (**a**) True star image; (**b**) blurred star image; (**c**) denoised star image with Bayes Shrink; (**d**) denoised star image with open operation; (**e**) denoised star image with the proposed algorithm.

In order to further test the processing effect of the proposed algorithm on the true star image, we use the star sensor with an integration time of 30 ms to obtain the star image. The star image under static condition is shown in Figure 12a and its resolution is 800 × 600. Under the dynamic condition of $w_{x}=w_{y}=4^{\circ }/s$, the image is blurred according to the parameters of the star sensor. Then the Bayes Shrink method, open operation, and the proposed algorithm are used, respectively, to denoise the image and the PSNRs of the denoised star images are obtained. In the proposed algorithm, α=1, the iteration number of the improved Gauss curvature filter is set to 3 and T is set to 50 according to the true intensity of the background noise. Experimental results indicate the PSNR of the original image is 27.671 and the PSNRs of the Bayes Shrink method, open operation, and the proposed algorithm are 32.914, 36.336, and 48.539, respectively. In addition, the SSIM (Structural Similarity Index) [18] between the denoised image using the proposed algorithm and the original image is 0.92, while the SSIMs of the Bayes Shrink method and the open operation are 0.83 and 0.77, respectively. The closer the SSIM is to 1, the more similar the denoised image is to original image. Thus, the proposed algorithm gives a much better denoising effect than that of the other two methods for a blurred star image.

### 5.2. Restoration of Blurred Star Image

**Figure 13:**
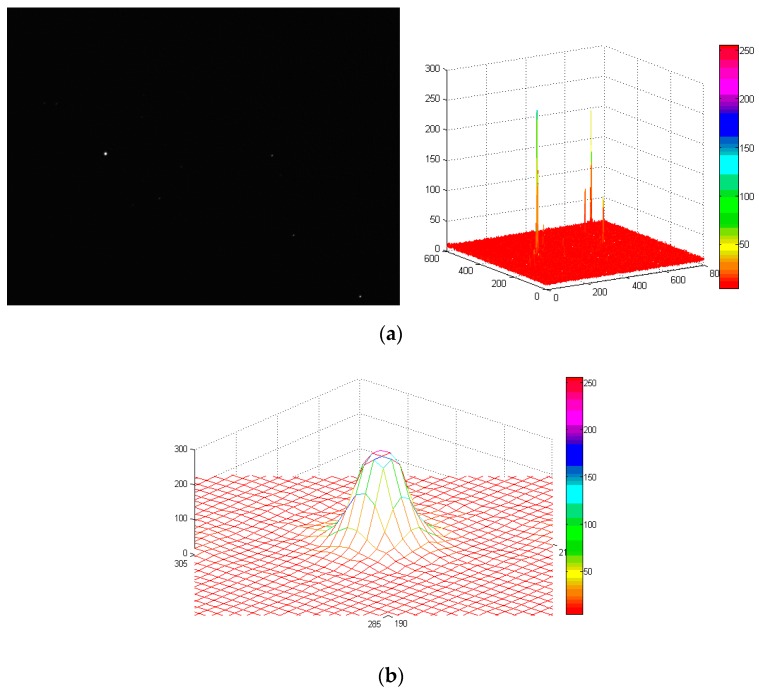
Restored star image of the proposed algorithm under the dynamic condition of $w_{x}=w_{y}=4^{\circ }/s$. (**a**) Star image restored by the proposed algorithm and its three-dimensional distribution; (**b**) energy distribution of NO.1 star.

**Figure 14:**
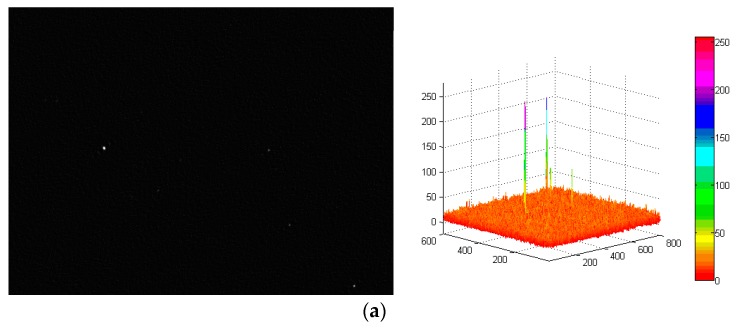

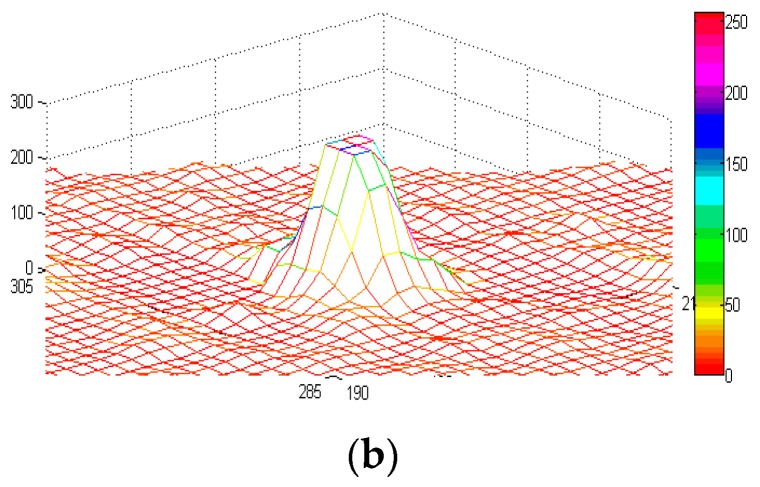
Star images of the Wiener filter under the dynamic condition of $w_{x}=w_{y}=4^{\circ }/s$. (**a**) Star image restored by Wiener filter and its three-dimensional distribution; (**b**) energy distribution of NO.1 star.

In order to verify the restoration performance of the proposed restoration algorithm, the true star image in Figure 12a is blurred under the dynamic conditions of $w_{x}=w_{y}=1^{\circ }/s$, $w_{x}=w_{y}=2^{\circ }/s$, and $w_{x}=w_{y}=4^{\circ }/s$. The blurred images are restored using the proposed algorithm and the Wiener filter [19], respectively. Then the centroid position in the restored star images are calculated, and the centroid error of the stars in restored star images can be obtained through the comparison with that in the true star image. Under the dynamic condition of $w_{x}=w_{y}=4^{\circ }/s$, the restored star images obtained using the proposed algorithm and the Wiener filter are shown in Figure 13 and Figure 14, respectively.

**Figure 15:**
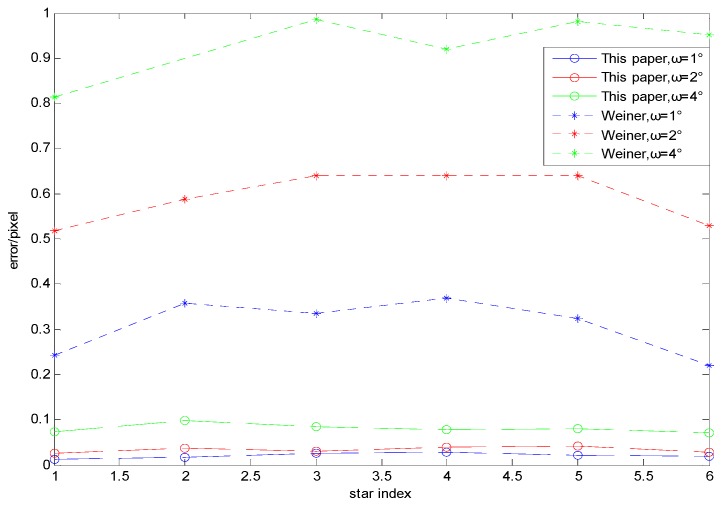
Errors of the star centroid coordinates for each method under different dynamic conditions.

Comparison of Figure 13 and Figure 14 reveals that under the same dynamic condition, in the image restored by the proposed algorithm, the background noise is less and the energy distribution of the star points is more uniform. With the Wiener filter, a ring effect of the star image occurs and the energy distribution of the star points is not uniform, which seriously decreases the accuracy of the determined centroid positions of the star points. To further illustrate the effectiveness of the restoration algorithm proposed in this paper, the centroid coordinates of stars under different angular velocities are determined. The calculated star centroid coordinates are shown in Table 3, and the error curve (taking the X axis error as an example) is shown in Figure 15.

The results show that with the increase of angular velocity, the centroid coordinates of all the stars in the image restored by the proposed algorithm can be located, and the centroid accuracies are within 0.1 pixels. However, as the angular velocity increases, the centroid accuracies of the stars in the image restored using the Wiener filter are highly decreased, which may lead to the failure of star extraction. For example, under the dynamic condition of $w_{x}=w_{y}=4^{\circ }/s$, NO.2 star is almost submerged by noise, making it impossible to extract the centroid coordinates of the stars. Therefore, compared with Wiener filtering, the determined centroid coordinates of the stars in the image processed by the proposed algorithm have higher accuracy.

Generally, when a star sensor works in dynamic conditions, the angular velocity of the star sensor is not constant, but continuously changing according to the actual working condition. Therefore, in order to further verify the proposed algorithm when the three-axis angular velocities of the star sensor vary simultaneously, an experiment is carried out. Four increasing angular velocity vectors are randomly selected in the range of $2\sim 4^{\circ }/s$, which represent four different dynamic conditions of the star sensor in four consecutive frames. The angular velocity vectors are shown in Table 4.

**Figure 16:**
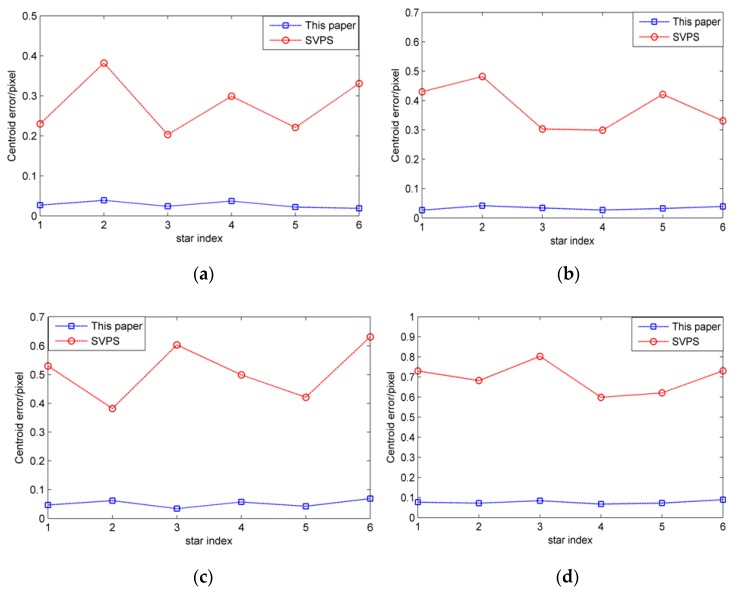
Star centroid errors under different angular velocity. (**a**) Star centroid errors in first frame; (**b**) star centroid errors in second frame; (**c**) star centroid errors in third frame; (**d**) star centroid errors in fourth frame.

Firstly, Figure 12a is used as the original star image in the first frame. Under the dynamic condition of index A, the original star image is blurred according to the parameters of the star sensor. Then, the proposed algorithm and SPVS (Space-Variant Point Spread) [20] algorithm are used to restore the blurred star image, and the centroid coordinates of each star in the restored star image are calculated by the centroid method. Finally, these centroid coordinates are compared with that of the corresponding stars in the original star image, and the centroid errors of the six stars in the first frame are obtained. Besides, the restored star image is used as the original star image in the next frame, and with the changed angular velocity vector, the experiment is repeated until the end of the fourth frame. The experimental results are shown in the Figure 16.

As shown in Figure 16, it can be seen that the larger the angular velocities are, the larger the centroid errors of the restored stars are. However, it is obvious that the restoration performance of the proposed algorithm is better than that of SVPS algorithm. With the proposed algorithm, the centroid errors are limited to 0.1 pixels, while the corresponding centroid errors of the SVPS algorithm are up to 0.9 pixels.

In order to verify the processing speed of the restoration algorithm in this paper, the processing time of the two algorithms are recorded in the process of restoration, and the results are shown in Table 5. As shown in Table 5, the processing time of our method is significantly less than that of SVPS, because the improved RL algorithm can terminate the iteration in time and has strong robustness.

## 6. Conclusions

Decreased SNR of the star image under dynamic condition reduces the accuracy of star centroid location. To address this limitation, a method based on an improved curvature filter is proposed to restore the blurred star image. First, the motion model of the star centroid is established and the degeneration function of the blurred star image under different working conditions are analyzed. Then an improved curvature filtering method based on energy function is presented to remove the noise and improve the SNR of the image. Besides, to improve the iteration process of the RL algorithm, the centroid coordinates of the stars in three consecutive frames of restored images are used to construct the termination condition of iteration equation. By using the proposed algorithm, the restoration effect of the blurred star image is improved and the accuracy of the star centroid location is increased. Denoising and restoration experiments are carried out under different dynamic conditions. Experimental results show that compared with the classic methods, the proposed algorithm can achieve better restoration effect and efficiency. Under the dynamic condition of $0\sim 4^{\circ }/s$, by using the proposed algorithm, the star centroid errors in the restored star image are within 0.1 pixels and the processing time of the star image is no longer than 103.054 ms, meeting the requirements of highly accurate and efficient star centroid location.

## Figures and Tables

**Table 1:** Parameter of star sensor.

Parameter	Value
FOV	6° × 4.5°
Focal length	60 mm
Pixel array	800 × 600 pixels
Integration time	60 ms
Detectable magnitude	6.5 Mv

**Table 2:** Signal-to-noise ration (PSNR) for each with various noise variance.

	Variance of Noise	Original Image	Bayes Shrink Method	Open Operation	Our Method
$w_{x}=w_{y}=1^{\circ }/s$	30	25.761	32.862	36.559	42.397
	50	23.608	29.538	35.668	40.621
	70	20.783	26.855	33.427	39.723
$w_{x}=w_{y}=2^{\circ }/s$	30	22.548	28.623	30.118	39.935
	50	20.833	25.477	28.822	36.842
	70	16.698	23.415	27.285	35.322
$w_{x}=w_{y}=4^{\circ }/s$	30	18.637	23.243	28.219	34.723
	50	17.824	22.945	27.563	32.463
	70	15.382	20.527	25.952	30.861

**Table 3:** Star centroid coordinates under different angular velocities.

Angular Velocity	Star Index	True Star Centroid	Wiener	Our Method
$w_{x}=w_{y}=1^{\circ }/s$	1	(199.484,294.782)	(199.727,295.037)	(199.471, 294.807)
	2	(358.863,319.762)	(359.221, 320.174)	(358.881, 319.742)
	3	(311.276,383.885)	(310.941, 384.272)	(311.301, 383.551)
	4	(541.497,299.656)	(541.866, 299.204)	(541.526, 299.627)
	5	(585.364,455.912)	(585.039, 455.577)	(585.385, 455.937)
	6	(719.756,582.563)	(719.975, 582.914)	(719.775, 582.585)
$w_{x}=w_{y}=2^{\circ }/s$	1	(199.484,294.782)	(200.001, 295.434)	(199.509, 295.03)
	2	(358.863,319.762)	(359.451, 319.334)	(358.901, 319.794)
	3	(311.276,383.885)	(310.635, 300.197)	(311.307, 383.91)
	4	(541.497,299.656)	(540.858, 298.971)	(541.536, 299.678)
	5	(585.364,455.912)	(586.005, 456.564)	(585.405, 455.96)
	6	(719.756,582.563)	(720.285, 583.114)	(719.785, 582.529)
$w_{x}=w_{y}=4^{\circ }/s$	1	(199.484,294.782)	(200.297, 293.895)	(199.411, 294.708)
	2	(358.863,319.762)	Fail	(358.961, 319.848)
	3	(311.276,383.885)	(312.261, 384.910)	(311.191, 383.811)
	4	(541.497,299.656)	(542.416, 300.512)	(541.418, 299.588)
	5	(585.364,455.912)	(586.345, 454.914)	(585.283, 455.837)
	6	(719.756,582.563)	(720.707, 583.559)	(719.685, 582.515)

**Table 4:** Angular velocity vectors of the star sensor.

Index	X Axis	Y Axis	Z Axis
A	2.4	2.2	2.6
B	2.9	2.8	2.9
C	3.3	3.5	3.2
D	3.8	3.9	3.8

**Table 5:** Processing time of restoration algorithms.

Frame Number	Our Method/ms	SVPS/ms
1	89.645	169.631
2	84.388	167.864
3	95.452	175.082
4	103.054	194.967
